# Longitudinal multi-b-value diffusion MRI for the early assessment of radiation-induced parotid injury in nasopharyngeal carcinoma

**DOI:** 10.3389/fonc.2026.1896045

**Published:** 2026-07-22

**Authors:** Lixuan Huang, Jifeng He, Hao Ren, Zongxiang Yang, Muliang Jiang, Jiayao Li, Yuqiu Nong, Apula Zuhere, Manyu Li, Mingjun Shen, Min Kang

**Affiliations:** 1 Department of Radiology, The First Affiliated Hospital of Guangxi Medical University, Nanning, Guangxi, China; 2 Department of Radiology, The Second Affiliated Hospital of Guangxi Medical University, Nanning, Guangxi, China; 3 Department of Radiology, Affiliated Hospital of Youjiang Medical University for Nationalities, Baise, Guangxi, China; 4 Department of Radiation Oncology, Affiliated Hospital of Youjiang Medical University for Nationalities, Baise, Guangxi, China; 5 Department of Radiation Oncology, The First Affiliated Hospital of Guangxi Medical University, Guangxi Tumor Radiation Therapy Clinical Medical Research Center, Nanning, Guangxi, China

**Keywords:** diffusion-weighted imaging, intensity-modulated radiotherapy, nasopharyngeal carcinoma, parotid damage, xerostomia

## Abstract

**Objective:**

Complications associated with nasopharyngeal carcinoma (NPC) treatment significantly impair patients’ quality of life. Radiotherapy-induced xerostomia is one of the most common complications of NPC treatment. This study aimed to evaluate the utility of quantitative parameters derived from DKI, CTRW, and FROC models for the early assessment of parotid gland damage following intensity-modulated radiotherapy (IMRT) in NPC. The findings may provide theoretical support for early identification of parotid injury and timely therapeutic intervention.

**Methods:**

A total of 54 patients with newly diagnosed NPC underwent multi-b-value DWI and routine MRI before radiotherapy, after 15 fractions, and at the end of IMRT. Parameters from the single-exponential DWI model, as well as from the DKI, CTRW, and FROC models, were calculated. Correlations among these parameters, IMRT dose, and xerostomia scores at the end of radiotherapy and 3 months after treatment were analyzed.

**Results:**

Most diffusion parameters derived from multi-b-value diffusion MRI of the parotid glands in patients with NPC gradually increased over the treatment period, whereas DKI_K values progressively decreased, with significant differences among time points (*P* < 0.05). The magnitude of parameter changes gradually decreased as treatment progressed. ΔDKI_K_Post-15RT and ΔDKI_K_Post-Pre were positively correlated with xerostomia scores at 3 months after IMRT (r=0.216 and 0.277; *P* = 0.012 and 0.002, respectively), whereas ΔCTRW_α_15RT-Pre and ΔCTRW_α_Post-Pre were negatively correlated (r=–0.258 and –0.232; *P* = 0.003 and 0.008, respectively). Statistically significant but weak correlations were found between several parameters and the planning target volume of nasopharyngeal carcinoma, minimum radiation dose (Dmin), and mean radiation dose (Dmean) (0.206≤|r|≤0.311, *P ≤* 0.05).

**Conclusion:**

Quantitative parameters derived from the single-exponential model, DKI, CTRW, and FROC enable dynamic detection of early microstructural alterations in the parotid glands after IMRT. The DKI_K and CTRW_α showed statistically significant but weak correlations with xerostomia scores in patients with nasopharyngeal carcinoma. These preliminary associations indicate that the two indices may serve as exploratory imaging biomarkers for evaluating radiation-induced parotid injury; however, their reliable predictive value requires further validation. In addition, dynamic changes in ADC, DKI_D, CTRW_α, FROC_β, FROC_D, and FROC_μ were correlated with radiation dose. These findings may provide preliminary evidence to support future investigations of individualized radiotherapy regimens.

## 1 Introduction

Nasopharyngeal carcinoma (NPC) is one of the most common malignant tumors in southern China. At present, the primary treatment approach for NPC is comprehensive therapy, with radiotherapy serving as the primary modality. However, radiation-induced parotid injury and subsequent xerostomia significantly impact both the quality of life and overall survival of patients with NPC (1). Although intensity-modulated radiotherapy (IMRT) has reduced such complications compared with conventional radiotherapy, outcomes remain suboptimal, and no effective treatment exists for established radiation-induced parotid damage (2, 3). Therefore, early assessment of xerostomia following radiotherapy is crucial for the timely diagnosis of parotid damage and for guiding adjustments to radiation dose (3).

Existing studies have demonstrated that imaging modalities such as B-mode ultrasound (4), computed tomography (CT) (5, 6), magnetic resonance imaging (MRI) (7), and salivary gland scintigraphy (8) are valuable tools for the early assessment of xerostomia. However, each modality has limitations for long-term or comprehensive monitoring. Color Doppler ultrasound evaluates salivary gland function by detecting hemodynamic changes resulting from spasms in the parotid microvasculature following radiotherapy. However, approximately 3 months after radiotherapy, cessation of radiation-induced stimulation leads to gradual recovery from vascular spasm, suggesting that Doppler ultrasound has limitations for long-term monitoring (4).

Multiparametric MRI (mpMRI) can detect early pathological microstructural changes and evaluate tissue architecture and cellular metabolism (9). As such, mpMRI has the potential to serve as a non-invasive alternative to ultrasound, CT, and salivary gland scintigraphy for predicting radiation-induced parotid gland damage. Conventional Gaussian diffusion models (DWI and DTI) assume unconfined, uniform water diffusion and cannot fully capture complex microstructural alterations in diseased tissues. Non-Gaussian models, including DKI, IVIM, CTRW, and FROC, have distinct advantages. Their physical assumptions closely reflect the restricted diffusion environment of biological tissues; they quantify deviations from Gaussian diffusion, thereby sensitively identifying subtle, heterogeneous microstructural damage in the early pathological phase. They provide multidimensional quantitative metrics to comprehensively characterize tissue damage. Several studies have proposed that mpMRI techniques, including intravoxel incoherent motion (IVIM) and three-dimensional pulsed continuous arterial spin labeling (ASL), may facilitate early detection and prediction of radiation-induced parotid injury by assessing individual microvascular perfusion and tissue diffusivity (10). Shen Mingjun et al. (11) reported a significant increase in IVIM parameter values from pre- to post-radiotherapy (*P* < 0.001), suggesting that IVIM may be useful for the dynamic monitoring of radiation-induced parotid gland damage.

Diffusion kurtosis imaging (DKI) enables quantification of diffusion kurtosis (K) and the diffusion coefficient (D). This technique can detect early, subtle tissue damage by capturing microstructural changes (12). Zhao et al. (13) reported that mean diffusivity, mean kurtosis, and their respective changes (ΔDKI_D, ΔDKI_K) derived from DKI were correlated with salivary flow rate and xerostomia questionnaire scores. These parameters were useful for tracking and monitoring acute damage to the parotid gland (PG), sublingual gland, and submandibular gland in NPC patients undergoing induction chemotherapy combined with concurrent chemoradiotherapy (IC+CCRT).

Fractional order calculus (FROC) and continuous-time random walk (CTRW) are advanced diffusion models used in multi-b-value DWI. They can capture both spatial and temporal heterogeneity in diffusion, offering a more accurate representation of the complex microstructure of biological tissues. Recent studies have demonstrated that the CTRW and FROC models are useful in lesion characterization (14), tumor grading (15), and prognosis prediction (16) across various cancer types. However, few reports have examined their application in assessing PG injury following intensity-modulated radiotherapy (IMRT) for nasopharyngeal carcinoma.

It remains unclear whether quantitative metrics from these advanced diffusion models can serve as imaging biomarkers for the early assessment of radiation-induced parotid gland injury or whether such parameters correlate with both radiation dose indices and clinical xerostomia severity among NPC patients receiving IMRT. This study aimed to investigate the potential clinical value of quantitative parameters derived from DKI, CTRW, and FROC models, based on multi-b-value non-Gaussian diffusion-weighted imaging, for the early assessment of PG damage in patients with NPC undergoing IMRT. The findings may provide a theoretical foundation for the early detection of parotid injury and timely intervention in clinical practice.

## 2 Materials and methods

### 2.1 Patients and methods

From June 2021 to September 2022, 54 patients with newly diagnosed NPC were prospectively enrolled. The inclusion criteria were as follows: (1) pathological confirmation of NPC by biopsy; (2) no contraindications to MRI; (3) no prior radiotherapy, chemotherapy, or other antitumor treatment; (4) a Karnofsky Performance Scale score greater than 70; (5) age 18 to 70 years; (6) receipt of conformal intensity-modulated radiotherapy following the initial MRI examination; and (7) voluntary participation in the study with written informed consent.

The exclusion criteria were as follows: (1) the presence of other malignant tumors; (2) a history of parotid disorders, including parotitis and parotid tumors; and (3) MRI images with severe artifacts.

This study was approved by the Institutional Review Board of the Ethics Committee of Guangxi Medical University (ethics approval number: 2022-KY-E-244). Written informed consent was obtained from all patients.

All patients received IMRT according to clinical staging and standard therapeutic principles. Among them, 3 patients (5.6%) underwent radiotherapy (RT) alone, 10 patients (18.5%) received CCRT, and the remaining 41 patients (75.9%) received IC+CCRT. The target volumes were defined according to the International Commission on Radiological Units and Measurements Report (ICRU) Nos. 50 (17) and 62 (18), and target-region delineation and radiotherapy planning were based on international clinical target delineation guidelines for nasopharyngeal carcinoma (19). The total IMRT dose ranged from 6,800 to 7,300 cGy, delivered in 30 to 33 fractions. Supportive symptomatic treatments, including antiemetics, gastric protection, and hepatoprotective therapy, were also administered concurrently. Radiation dosimetric parameters were obtained separately from the dose-volume histograms generated by the treatment planning system, including the planning target volume for the nasopharynx (PTVnx), minimum dose to the bilateral parotid glands (Dmin), mean dose to the bilateral parotid glands (Dmean), and maximum dose to the bilateral parotid glands (Dmax).

The collected clinical data included patient age, sex, pathological tumor type, TN stage, and details of radiotherapy and chemotherapy. Staging was determined according to the 8th edition of the UICC/AJCC nasopharyngeal carcinoma staging system based on findings from pretreatment MRI, CT, and PET-CT examinations.

### 2.2 Imaging protocol

A fully digital 3.0 T MR scanner (Prisma, Siemens, Germany) with a 20-channel head/neck phased-array coil was used for the MR examinations. Each patient underwent three MRI and DWI sessions: 2 weeks before radiotherapy (pre-RT), after 15 fractions of radiotherapy (mid-RT/15RT), and after radiotherapy (post-RT). Patients were scanned in the supine position while breathing freely. The scan range extended from the middle temporal lobe to the thoracic inlet. To minimize motion artifacts, patients were instructed to limit swallowing and remain as still as possible during the examination. The scanning sequences included axial T1WI, axial T2WI, axial T2WI_FS, and axial multi-b-value diffusion MRI. The multi-b-value diffusion MRI scan parameters were as follows: slice thickness = 3 mm; interslice spacing = 0 mm; TR/TE = 4000/61 ms; FOV = 200 x 200 mm; number of excitations = 2; b-values = 0, 10, 20, 30, 50, 100, 150, 200, 600, 1000, 1500, and 2000 s/mm2; and scanning time = 5 min 10 s.

### 2.3 Image analysis

The original multi-b-value DWI DICOM images were imported into the postprocessing software Body Diffusion Lab (BoDiLab) to generate parameter maps from the DWI, DKI, CTRW, and FROC models.

The fitting formula for the single-exponential DWI model is defined as follows:


Sb/S0=exp(−b×ADC)

S0 and Sb represent the signal intensities acquired at b=0 s/mm^2^ and b=1000 s/mm^2^, respectively. The unit of the apparent diffusion coefficient (ADC) is 10–^6^ mm^2^/s.

The fitting formula for the DKI model is as follows:


$$Sb/S0=\text{exp}(-bD+1/6b^{2}D^{2}K)$$

D and K are fitting variables. Sb denotes the signal intensity at a specific b-value, and S0 corresponds to the signal intensity without diffusion weighting (b=0 s/mm^2^). K represents diffusion kurtosis, and D represents the diffusion coefficient; both are corrected for non-Gaussian water diffusion during fitting. The kurtosis parameter quantifies the deviation of water molecular motion from ideal Gaussian diffusion; kurtosis equals zero for purely Gaussian diffusion, whereas larger kurtosis values indicate more pronounced non-Gaussian diffusion behavior. Parametric maps of D and K were automatically generated for each patient using the postprocessing software.

The fitting formula for the CTRW model is shown below:


$$Sb/S0=E_{\alpha }[-{\left(bD\right)}^{\beta }]$$

Sb refers to the signal intensity under a diffusion weighting factor of b, and S0 is the signal intensity without diffusion weighting. Eα denotes the Mittag-Leffler function of order α, and β represents the spatial fractional derivative reflecting spatial diffusion heterogeneity. Both α and β range from 0 to 1; α=1 and β=1 for homogeneous diffusion media. Parametric maps of D, α, and β were automatically exported for each patient.

The fitting formula for the FROC model is as follows:


$$Sb/S0=exp[-D\mu^{2(\beta -1)}{\left(\gamma \;Gd\;\delta \right)}^{2\beta }(\Delta -\frac{2\beta -1}{2\beta +1}\;\delta )]$$

Sb denotes the signal intensity at diffusion weighting factor b, and S0 is the unweighted baseline signal. Gd, δ, and Δ correspond to diffusion gradient amplitude, gradient pulse duration, and gradient separation interval, respectively. D is the diffusion coefficient with units of μm^2^/ms; β is the dimensionless spatial fractional derivative (0<β≤1); μ is a spatial parameter measured in μm. The postprocessing software automatically generated parametric maps of D, β, and μ for all enrolled patients.

**Figure 1:**
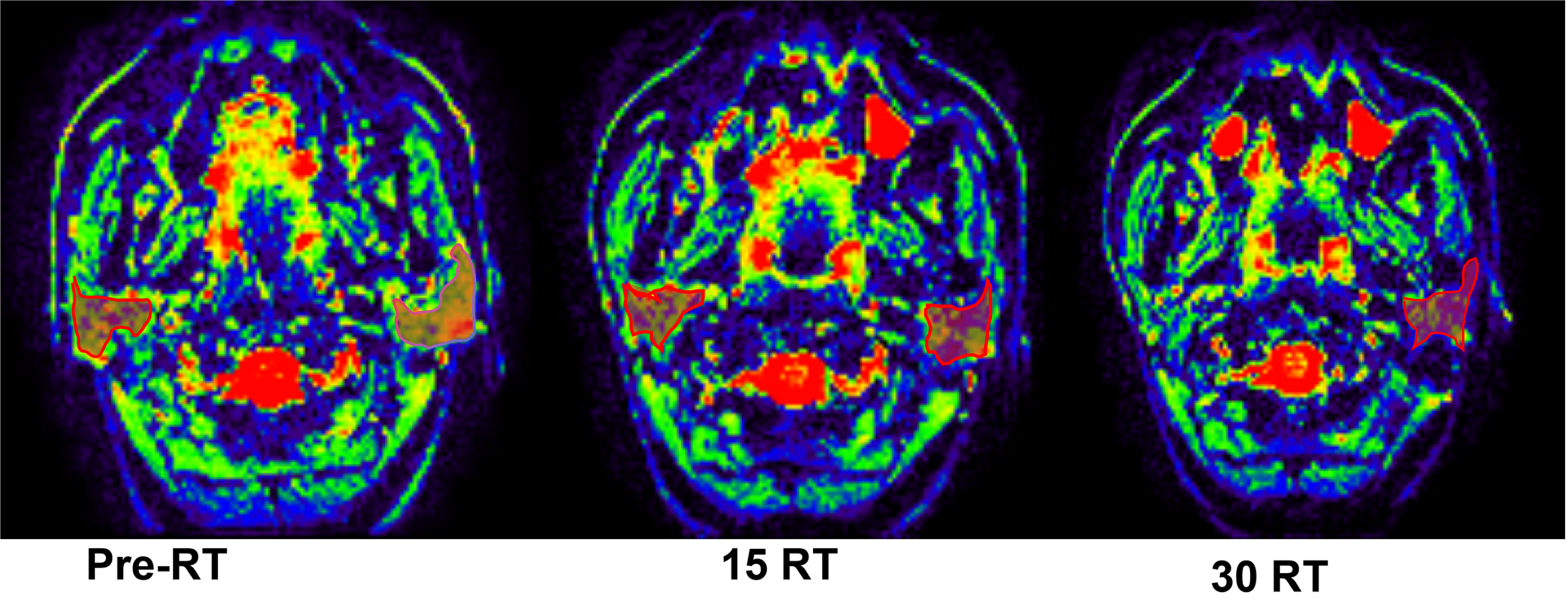
The ROI was shown as a red semitransparent irregular region.

Regions of interest (ROIs) were manually delineated independently by two radiologists with more than 10 years of experience in head and neck MRI interpretation. Any disagreements between the two physicians were resolved through discussion until consensus was reached. Using axial T2WI and contrast-enhanced sequences as anatomical references, ROIs were drawn on the S0 images of the DKI sequence at the largest cross-sectional area of the PG and at two consecutive levels above and below this section, while carefully excluding blood vessels, nerves, and adipose tissue (**Figure 1**). The mean value from these three slices was used as the final measurement. The defined ROIs were then automatically mapped to all corresponding parametric maps within the same anatomical layers to compute parameter values. As a result, the following parameters were obtained for each NPC patient: DKI_K, DKI_D, ADC, CTRW_D, CTRW_α, CTRW_β, FROC_D, FROC_β, and FROC_μ.

### 2.4 Xerostomia assessment

According to the RTOG/EORTC radiation injury grading criteria (20), radiation-induced xerostomia was scored by two independent radiation oncologists before MRI examinations at the end of radiotherapy and 3 months after treatment to assess its severity. Both clinicians were completely blinded to all diffusion MRI parametric maps and radiotherapy dosimetric data during subjective scoring to avoid assessment bias.

### 2.5 Statistical analysis

Statistical analyses were performed using SPSS version 26.0. Categorical variables were reported as frequencies. The normality of each dataset was evaluated using the Kolmogorov-Smirnov test before group comparisons. Continuous data with a normal distribution were expressed as mean ± standard deviation (SD), whereas non-normally distributed data were summarized as median with interquartile range (IQR). For paired longitudinal comparisons, repeated-measures ANOVA was used for normally distributed data, whereas the Friedman test was used for non-normally distributed data. When significant differences were identified, pairwise comparisons were performed using the Bonferroni *post hoc* test. One-way ANOVA was used to compare multiple groups. Benjamini-Hochberg false discovery rate (FDR) correction was applied to all pairwise inter-timepoint comparisons of MRI quantitative parameters and their dynamic variations, and adjusted q-values were used to determine statistical significance (q < 0.05). Intraclass correlation coefficients (ICCs) with 95% confidence intervals (CIs) were used to assess inter-reader agreement for the quantitative measurements. Spearman’s correlation analysis was conducted to evaluate the relationships between each quantitative parameter and both the conformal intensity-modulated radiation dose and the xerostomia score. Correlation coefficients were interpreted as follows: 0 ≤ |r| <0.20, no correlation; 0.20 ≤ |r| <0.40, weak correlation; 0.40 ≤ |r| <0.60, moderate correlation; 0.60 ≤ |r| <0.80, strong correlation; and 0.80 ≤ |r| ≤ 1.00, extremely strong correlation. Mixed-effects models with patient random intercepts were constructed to evaluate temporal effects by separately entering quantitative MRI parameters of the left and right parotid glands and patient-level data for both parotid glands.

## 3 Results

### 3.1 Patients

Demographic and clinical data for the 54 enrolled patients (34 males and 20 females; mean age, 45.09 ± 11.86 years) are presented in **Table 1**. All patients received a planned IMRT regimen and underwent nasopharyngeal MRI examinations at three time points: 2 weeks before treatment, after 15 radiotherapy fractions, and upon completion of radiotherapy.

**Table 1:** Clinical characteristics of patients included in this study.

Characteristic	NPC (n=54)
Age (years)	45.09 ± 11.86
Sex	
Male	34
Female	20
WHO pathological type	
I	0
IIa	2
IIb	37
III	2
T stage	
T1	1
T2	6
T3	24
T4	23
N stage	
N0	0
N1	16
N2	16
N3	22
Clinical stage	
II	3
III	14
IVA	35
IVB	2
Treatment	
IC+CCRT	41
CCRT	10
RT	3
Xerostomia grade (before RT)	
Grade 0	54

### 3.2 PGs MRI quantitative parameter analysis

#### 3.2.1 Changes in MRI quantitative parameters of the parotid glands

**Figure 2:**
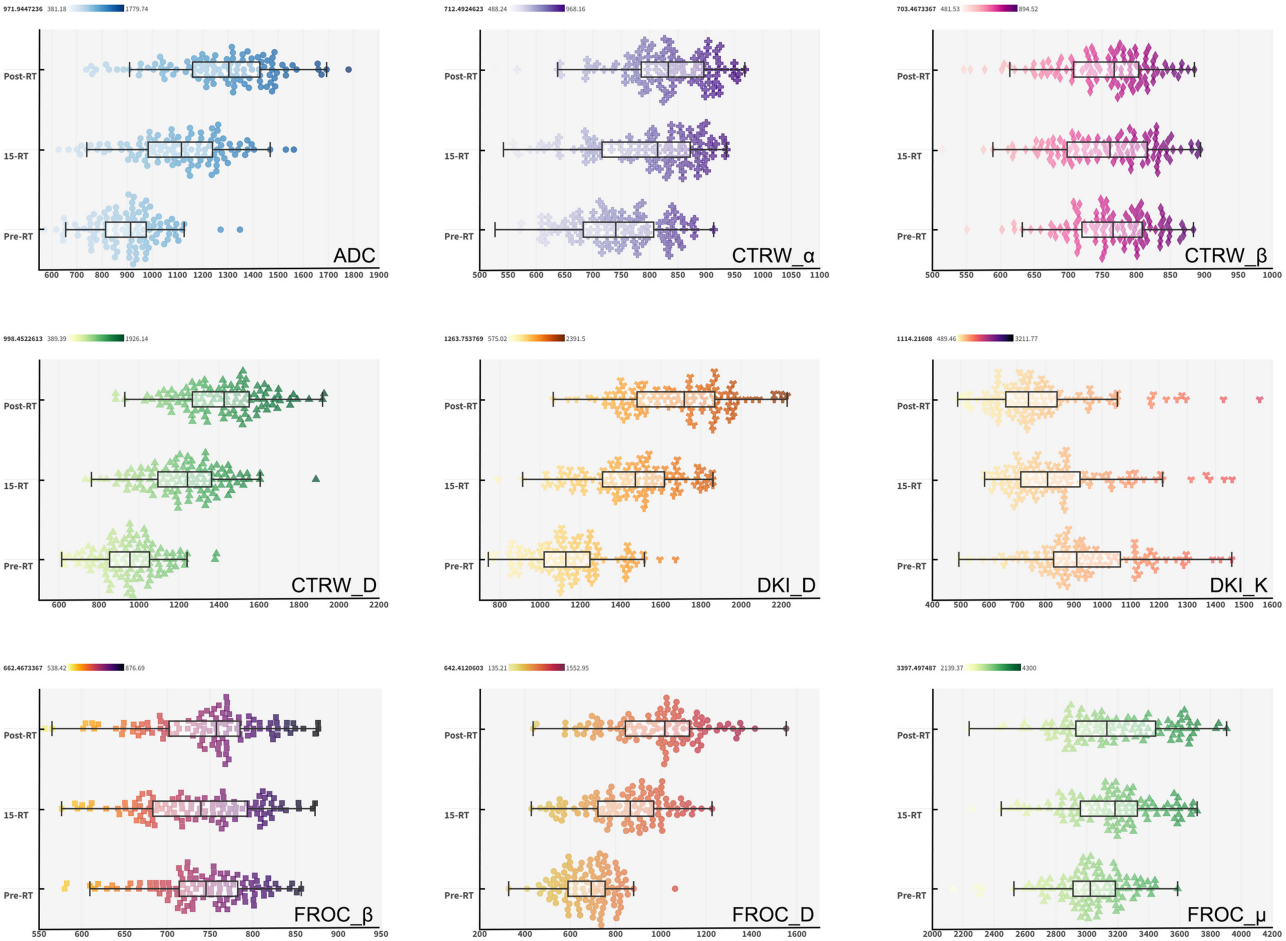
Comparison of parameters at different time points during radiotherapy. D and ADC: × 10–^6^ mm^2^/s; FROC_μ: × 10–^6^ mm.

The interobserver ICC values for all DKI, CTRW, and FROC quantitative parameters ranged from 0.67 to 0.91, indicating good reproducibility of manual ROI delineation. Parameter values derived from the various DWI models for 108 parotid glands in 54 NPC patients, as well as their changes over time, are presented in **Figure 2** and **Table 2**. As the duration of radiotherapy increased, the values of ADC, DKI_D, CTRW_D, CTRW_α, FROC_D, FROC_β, and FROC_μ gradually increased, whereas DKI_K decreased. Statistically significant differences (*P* < 0.05) were observed in ADC, DKI_D, CTRW_D, CTRW_α, FROC_D, and DKI_K across the three time points: before radiotherapy, after 15 radiotherapy fractions, and at the end of radiotherapy. In addition, FROC_μ values showed statistically significant differences between pre-radiotherapy measurements and those obtained after 15 fractions and at the end of radiotherapy (*P* < 0.05).

**Table 2:** Comparison of parameters at different treatment time points.

Parameter	Pre (n=108)	15RT (n=108)	Post (n=108)			
	Mean value	Mean value	Mean value	P1	P2	P3
ADC	897.08 ± 142.86	1102.35 ± 189.99	1267.55 ± 219.17	0.001^b^	0.000^b^	0.001^a^
DKI_D	1135.68 ± 201.84	1461.07 ± 249.19	1684.31 ± 250.90	0.000^b^	0.000^b^	0.001^a^
DKI_K	916.07(284.73)	812.68(237.61)	742.93(192.89)	0.001^b^	0.001^b^	0.004^b^
CTRW_D	949.09 ± 165.23	1229.06 ± 200.18	1414.77 ± 215.54	0.000^b^	0.000^b^	0.001^a^
CTRW_α	742.40 ± 81.62	814.19(157.61)	832.41(114.79)	0.001^a^	0.001^b^	0.025^b^
CTRW_β	764.12 ± 63.75	751.34 ± 81.24	767.41(97.416)	0.325^b^	0.300^b^	0.936^b^
FROC_D	671.47 ± 126.71	843.58 ± 177.26	1017.24(285.70)	0.001^b^	0.000^b^	0.001^b^
FROC_β	739.89 ± 61.10	737.11 ± 67.28	757.25(86.05)	0.736^a^	0.434^b^	0.299^b^
FROC_μ	3028.98(278.41)	3154.87 ± 300.04	3170.90 ± 341.98	0.001^b^	0.004^b^	0.715^a^

^a^Repeated-measures ANOVA was used to perform statistical analyses; ^b^Wilcoxon signed-rank test was used to perform statistical analyses. Pre: pre-radiotherapy; 15RT: after 15 fractions of radiotherapy; Post: end of radiotherapy. *P^1^*: pre-radiotherapy versus after 15 fractions of radiotherapy; *P^2^*: pre-radiotherapy versus the end of radiotherapy; *P^3^*: after 15 fractions of radiotherapy versus the end of radiotherapy. All parameters: D and ADC, × 10–^6^ mm^2^/s; FROC_μ, × 10–^6^ mm. Normally and non-normally distributed data are presented as mean ± SD and median (IQR), respectively.

We further constructed mixed-effects models incorporating patient random intercepts to assess temporal effects. The results showed consistent effect directions and statistical significance for ADC, DKI_D, DKI_K, CTRW_D, CTRW_α, and FROC_D between the left-sided and right-sided models. At the patient level, ADC, DKI_D, CTRW_D, CTRW_α, and FROC_D showed the same direction of effect and statistical significance as in the left-only and right-only models. The main conclusions remained unchanged when analyses were restricted to either left or right parotid data, indicating that the study findings were robust in the side-based sensitivity analysis. These results are presented in **Supplementary Tables 2**-**5**.

#### 3.2.2 Dynamic changes in MRI quantitative parameters of parotid glands

The dynamic changes in MRI parameters between the end of radiotherapy and after 15 radiotherapy fractions (ΔADC_Post-15RT, ΔDKI_D_Post-15RT, ΔCTRW_D_Post-15RT, ΔCTRW_α_Post-15RT, and ΔFROC_D_Post-15RT) were significantly lower than those observed between pre-radiotherapy and after 15 fractions (ΔADC_15RT-Pre, ΔDKI_D_15RT-Pre, ΔCTRW_D_15RT-Pre, ΔCTRW_α_15RT-Pre, and ΔFROC_D_15RT-Pre). This finding suggests that the rate of increase in parameter values slowed as treatment progressed. In addition, ΔDKI_K showed a consistent downward trend, whereas ΔFROC_μ, ΔFROC_β, and ΔCTRW_β showed no clear trends (**Table 3**).

**Table 3:** Comparison of dynamic parameter changes at different treatment time points.

Parameters	15RT-Pre (n=108)	Post-Pre (n=108)	Post-15RT (n=108)	P1	P2	P3
	Mean value	Mean value	Mean value			
ΔADC	205.28 ± 181.76	397.24(290.43)	165.20(290.43)	0.000^b^	0.020^b^	0.000^b^
ΔDKI_D	325.40 ± 258.65	548.63 ± 293.91	226.34(251.20)	0.001^b^	0.001^b^	0.001^b^
ΔDKI_K	-116.11(265.98)	-150.37(239.69)	-64.23(161.57)	0.036^b^	0.109^b^	0.000^b^
ΔCTRW_D	279.97(161.57)	465.68 ± 229.05	181.07(190.87)	0.001^b^	0.001^b^	0.000^b^
ΔCTRW_α	51.88 (113.23)	82.20(92.95)	31.66(92.95)	0.008^b^	0.036^b^	0.000^b^
ΔCTRW_β	-6.01(93.40)	-13.08 ± 73.47	-0.30 ± 81.97	0.918^b^	0.238^b^	0.194^a^
ΔFROC_D	187.38 (179.49)	313.62 ± 178.75	142.81(143.82)	0.000^a^	0.029^b^	0.000^b^
ΔFROC_β	0.34(74.98)	4.77 ± 66.09	7.55 ± 70.80	0.344^b^	0.328^b^	0.744^a^
ΔFROC_μ	170.93(425.14)	142.53 ± 423.45	16.035(425.1)	0.765^b^	0.030^b^	0.016^a^

^a^Repeated-measures ANOVA was used to perform statistical analyses; ^b^Wilcoxon signed-rank test was used to perform statistical analyses. 15RT-Pre: dynamic changes between pre-radiotherapy and 15 fractions of radiotherapy; Post-Pre: dynamic changes between pre-radiotherapy and the end of radiotherapy; Post-15RT: dynamic changes between 15 fractions of radiotherapy (mid-RT/15RT) and the end of radiotherapy. *P^1^*: 15RT-Pre versus Post-Pre; *P^2^*: 15RT-Pre versus Post-15RT; *P^3^*: Post-Pre versus Post-15RT. All parameters: D and ADC, × 10–^6^ mm^2^/s, FROC_μ: × 10–^6^ mm. Normally and non-normally distributed data are presented as mean ± SD and median (IQR), respectively.

After FDR correction, consistently significant inter-timepoint differences (all q < 0.001) remained detectable for ADC, DKI_D, DKI_K, CTRW_D, CTRW_α, and FROC_D, whereas CTRW_β and FROC_β showed no statistically significant differences (all q > 0.05). The Post-Pre changes in DKI_D, DKI_K, CTRW_D, CTRW_α, and FROC_D differed significantly from the Post-15RT changes, and the 15RT-Pre changes in DKI_D and CTRW_D also differed significantly from the Post-15RT changes (all q < 0.001). These results showed that adjustment for multiple comparisons did not change the main positive conclusions while reducing the risk of false positives (**Supplementary Tables 6**, **7**).

### 3.3 Correlation between MRI quantitative parameters of the parotid gland and Xerostomia score

At the end of radiotherapy, 11, 37, and 6 patients presented with Grade 1, Grade 2, and Grade 3 xerostomia, respectively. At 3 months after radiotherapy, the numbers of patients with Grade 0, Grade 1, Grade 2, and Grade 3 xerostomia were 1, 20, 32, and 1, respectively. Among the parameter changes across different radiotherapy stages, ΔDKI_K_Post-15RT and ΔDKI_K_Post-Pre were positively correlated with xerostomia scores at 3 months post-radiotherapy (r=0.216 and 0.277; *P* = 0.012 and 0.002, respectively). In contrast, ΔCTRW_α_15RT-Pre and ΔCTRW_α_Post-Pre were negatively correlated with xerostomia scores at the same time point (r=–0.258 and –0.232, *P* = 0.003 and 0.008, respectively), as shown in **Table 4**. However, no significant correlations were observed between parameter changes and xerostomia scores at the end of radiotherapy or between individual MRI parameter values at each radiotherapy stage and xerostomia scores at 3 months post-treatment.

**Table 4:** Correlation between dynamic MRI quantitative parameters and xerostomia score at 3 months.

Parameters	15RT-Pre (n=108)		Post-15RT (n=108)		Post-Pre (n=108)	
	r	P	r	P	r	P
ΔADC	-0.115	0.118	-0.117	0.114	-0.177	0.032
ΔDKI_D	-0.062	0.263	-0.066	0.25	-0.127	0.094
ΔDKI_K	0.136	0.08	0.216	0.012	0.277	0.002
ΔCTRW_D	-0.03	0.38	-0.057	0.28	-0.094	0.166
ΔCTRW_α	-0.258	0.003	0.063	0.258	-0.232	0.008
ΔCTRW_β	0.028	0.385	-0.197	0.02	-0.147	0.065
ΔFROC_D	-0.054	0.288	-0.162	0.047	-0.159	0.05
ΔFROC_β	0.007	0.471	-0.196	0.021	-0.188	0.026
ΔFROC_μ	-0.09	0.176	0.125	0.099	0.07	0.236

Spearman’s correlation was used to assess correlations between dynamic MRI quantitative parameters and xerostomia score at 3 months, with results reported as r (*P*). 15RT-Pre: dynamic changes between pre-radiotherapy and 15 fractions of radiotherapy; Post-15RT: dynamic changes between 15 fractions of radiotherapy (mid-RT/15RT) and the end of radiotherapy; Post-Pre: dynamic changes between pre-radiotherapy and the end of radiotherapy.

### 3.4 Correlation analysis of MRI quantitative parameters with radiation dose

#### 3.4.1 Correlation analysis of MRI quantitative parameters with radiation dose

The correlations between quantitative imaging parameters and radiation dose at different radiotherapy stages in parotid tissue are presented in **Table 5**. The results demonstrated statistically negative correlations between FROC_D and FROC_μ values after 15 fractions of radiotherapy and PTVnx (r=-0.249 and -0.251, respectively; *P* = 0.009 for both).

**Table 5:** Correlation analysis of quantitative parameters at different time points with radiation dose.

Parameters	r(P)				
		PTVnx	Dmin	Dmax	Dmean
FROC_D	Pre	-0.130(0.179)	0.168(0.082)	-0.096(0.325)	0.026(0.787)
	15 RT	-0.249(0.009)	0.006(0.954)	0.020(0.840)	-0.017(0.860)
	Post	-0.114(0.239)	-0.050(0.607)	-0.005(0.959)	0.027(0.780)
FROC_μ	Pre	-0.062(0.521)	0.110(0.258)	-0.103(0.287)	-0.042(0.667)
	15 RT	-0.251(0.009)	-0.014(0.888)	-0.016(0.866)	-0.033(0.732)
	Post	-0.076(0.434)	-0.033(0.733)	0.003(0.978)	0.043(0.662)

Spearman’s correlation was used to assess correlations between quantitative parameters at different time points and radiation dose, with results reported as r (*P*). Pre: pre-radiotherapy; 15RT: after 15 fractions of radiotherapy; Post: end of radiotherapy; PTVnx: planning target volume for the nasopharynx; Dmin: planned minimum dose to the parotid gland; Dmean: planned mean dose to the parotid gland; Dmax: planned maximum dose to the parotid gland.

#### 3.4.2 Correlation analysis of quantitative parameter dynamic changes with radiation dose

As shown in **Table 6**, the correlations between changes in quantitative imaging parameters and radiation dose at different stages of radiotherapy were analyzed. Specifically, ΔADC_Post-Pre, ΔADC_Post-15RT, ΔDKI_D_Post-Pre, ΔDKI_D_Post-15RT, ΔCTRW_α_15RT-Pre, ΔCTRW_α_Post-Pre, ΔFROC_β_15RT-Pre, ΔFROC_β_Post-Pre, and ΔFROC_μ_15RT-Pre were all negatively correlated with PTVnx (r=-0.277, -0.219, -0.273, -0.235, -0.206, -0.311, -0.219, -0.265, and -0.222; *P* = 0.004, 0.023, 0.004, 0.015, 0.033, 0.001, 0.023, 0.006, and 0.021, respectively). In addition, ΔFROC_D_Post-Pre was negatively correlated with Dmin (r=-0.208, *P* = 0.031). Moreover, ΔADC_15RT-Pre, ΔDKI_D_15RT-Pre, ΔCTRW_α_15RT-Pre, and ΔCTRW_α_Post-Pre showed statistically positive correlations with Dmean (r=0.220, 0.209, 0.239, and 0.207; *P* = 0.022, 0.030, 0.013, and 0.032, respectively).

**Table 6:** Correlation analysis of dynamic changes in quantitative parameters between different radiotherapy stages and radiation dose.

Parameters	r(P)				
		PTVnx	Dmin	Dmax	Dmean
ΔADC	15RT-Pre	-0.118 (0.223)	-0.068(0.486)	0.092(0.341)	0.220(0.022)
	Post-Pre	-0.277(0.004)	0.020(0.840)	0.028(0.775)	0.192(0.047)
	Post-15RT	-0.219(0.023)	0.093(0.337)	0.009(0.927)	0.001(0.996)
ΔDKI_D	15RT-Pre	-0.124(0.200)	-0.045(0.646)	0.052(0.594)	0.209(0.030)
	Post-Pre	-0.273(0.004)	0.059(0.542)	0.033(0.734)	0.193(0.045)
	Post-15RT	-0.235(0.015)	0.095(0.326)	0.031(0.747)	-0.008(0.932)
ΔDKI_K	15RT-Pre	0.026(0.786)	-0.039(0.686)	-0.164(0.090)	-0.159(0.101)
	Post-Pre	0.088(0.364)	0.080(0.408)	-0.106(0.276)	-0.155(0.109)
	Post-15RT	0.043(0.6560	0.040(0.685)	0.045(0.640)	-0.023(0.814)
ΔCTRW_α	15RT-Pre	-0.206(0.033)	-0.112(0.248)	0.108(0.268)	0.239(0.013)
	Post-Pre	-0.311(0.001)	0.012(0.906)	0.050(0.605)	0.207(0.032)
	Post-15RT	-0.183(0.058)	0.090(0.357)	0.006(0.947)	-0.042(0.665)
ΔCTRW_β	15RT-Pre	-0.028(0.775)	0.113(0.246)	0.041(0.673)	0.108(0.267)
	Post-Pre	-0.128(0.186)	0.159(0.100)	-0.010(0.915)	0.071(0.466)
	Post-15RT	-0.117(0.227)	0.036(0.714)	0.005(0.963)	0.003(0.977)
ΔFROC_D	15RT-Pre	-0.171(0.078)	-0.102(0.293)	0.096(0.325)	-0.020(0.838)
	Post-Pre	0.031(0.749)	-0.208(0.031)	0.051(0.597)	-0.047(0.632)
	Post-15RT	0.165(0.087)	-0.066(0.496)	-0.026(0.786)	-0.004(0.967)
ΔFROC_β	15RT-Pre	-0.219(0.023)	-0.112(0.249)	0.082(0.400)	0.188(0.051)
	Post-Pre	-0.265(0.006)	0.008(0.932)	0.023(0.814)	0.184(0.057)
	Post-15RT	-0.139(0.150)	0.069(0.476)	-0.028(0.772)	-0.027(0.781)
ΔFROC_μ	15RT-Pre	-0.222(0.021)	-0.070(0.470)	0.101(0.300)	0.073(0.455)
	Post-Pre	0.002(0.981)	-0.154(0.112)	0.095(0.327)	0.042(0.663)
	Post-15RT	0.196*(0.042)	-0.058(0.553)	0.021(0.831)	0.038(0.695)

Spearman’s correlation was used to assess correlations between dynamic changes in quantitative parameters and radiation dose, with results reported as r (*P*). 15RT-Pre: dynamic changes between pre-radiotherapy and 15 fractions of radiotherapy; Post-Pre: dynamic changes between pre-radiotherapy and the end of radiotherapy; Post-15RT: dynamic changes between 15 fractions of radiotherapy (mid-RT/15RT) and the end of radiotherapy. PTVnx: planning target volume for the nasopharynx; Dmin: planned minimum dose to the parotid gland; Dmean: planned mean dose to the parotid gland; Dmax: planned maximum dose to the parotid gland.

## 4 Discussion

This study used quantitative parameters derived from multi-b-value non-Gaussian DWI models, specifically DKI, CTRW, and FROC, to reflect structural heterogeneity directly and dynamically assess early microstructural damage to the PG induced by IMRT. Notably, the CTRW model offers a novel method for characterizing tissue and environmental heterogeneity by quantifying diffusion time and spatial complexity through the α and β parameters on a voxel-wise basis. Our findings revealed that quantitative parameters from the mono-exponential model, as well as from the DKI, CTRW, and FROC models, exhibited characteristic trends as radiation dose increased during radiotherapy in NPC patients. Among these, ADC, DKI_K, CTRW_D, CTRW_α, FROC_D, and FROC_μ emerged as the most critical parameters. Furthermore, correlation analysis demonstrated statistically significant but weak correlations between several multiparametric functional MRI parameters and both xerostomia scores and radiation dose distribution metrics. These quantitative diffusion parameters may provide supplementary imaging evidence to support objective xerostomia evaluation in NPC patients after radiotherapy.

In recent years, as survival rates among patients with NPC have improved, increasing emphasis has been placed on quality of life. Consequently, early prediction of xerostomia following radiotherapy and chemotherapy, along with timely adjustment of treatment plans to minimize PG dysfunction, has become a research focus of considerable clinical importance (3). Previous studies have highlighted early PG damage using ASL and IVIM imaging, which primarily assess microperfusion and thus indirectly reflect changes in tissue microstructure. For example, by evaluating dynamic changes in IVIM and ASL parameters during early-stage PG treatment in NPC patients, Zhang et al. suggested that these techniques may aid in the early detection and prediction of radiation-induced parotid injury following radiotherapy (10).

In addition, other studies have investigated post-radiotherapy changes in ADC values, suggesting that DWI may be a valuable tool for detecting physiological alterations in the salivary glands of NPC patients undergoing radiotherapy (9, 21, 22). In this study, ADC gradually increased during intensity-modulated radiotherapy (IMRT), with significant differences among the pretreatment, 15-fraction, and post-treatment time points, while the rate of ADC increase slowed over time; these findings are consistent with previous reports (23, 24). The observed increase in ADC values may be attributed to increased extracellular water content and reduced cell density within the PG, likely due to radiation-induced apoptosis, necrosis of glandular cells, and tissue fibrosis. The slowing trend in ADC elevation may be associated with early stages of PG repair (25).

Compared with conventional DWI, DKI better characterizes non-Gaussian water diffusion (26, 27). In this study, DKI_K value decreased, and DKI_D value increased continuously during radiotherapy, which may be attributable to lymphocyte infiltration, interstitial edema, and increased capillary permeability in the PG. These findings may reflect significant alterations in the diffusion characteristics of water molecules within parotid tissue following radiotherapy and are consistent with previous results (28). We also observed that the rate of increase in DKI_D values slowed over time, which may be associated with attenuated PG tissue damage, acinar cell regeneration, and the initiation of reparative processes within the gland.

The increase in CTRW_D observed in this study suggests that water diffusion became progressively less restricted, further corroborating the changes in ADC, DKI_D, and DKI_K. In addition, the increase in CTRW_α during radiotherapy indicated heightened tissue heterogeneity, potentially reflecting extensive damage to parotid acinar cells. FROC_D is considered a more accurate quantitative index of tissue diffusivity. In this study, the change in FROC_D followed a trend consistent with that of ADC. Previous studies have shown that FROC_μ is negatively correlated with the mean free path of diffusing molecules (29). In this study, FROC_μ also increased with the progression of radiotherapy, suggesting a compensatory angiogenic response and cellular reorganization as part of the gland’s early repair mechanisms following injury.

Integrating imaging findings with xerostomia scores enables a more objective evaluation of xerostomia grade. This study found that both ΔDKI_K_Post-15RT and ΔDKI_K_Post-Pre exhibited mild positive correlations with xerostomia scores at 3 months following IMRT. In other words, greater changes in DKI_K values were associated with higher xerostomia scores, indicating more severe dry mouth symptoms. This may be attributable to radiation-induced damage to salivary gland acinar cells (30). In addition, this study found that ΔCTRW_α_15RT-Pre and ΔCTRW_α_Post-Pre were mildly negatively correlated with xerostomia scores at 3 months after IMRT. Smaller changes in CTRW_α, namely lower variation in diffusion-related parameters, may be associated with poor tissue repair capacity and a reduction in the number of surviving acinar cells, which, in turn, leads to greater xerostomia severity. These findings suggest that the pathological and functional alterations of the PG following radiation injury partially reflect the subjective clinical grading of xerostomia. Notably, the detected correlations between parotid diffusion biomarkers and xerostomia severity were weak in magnitude. Although these associations reached statistical significance, this significance merely indicates a mild linear trend rather than guaranteeing strong clinical predictive power. Collectively, variations in DKI_K and CTRW_α weakly mirrored post-radiotherapy xerostomia severity and dynamically reflected parotid gland functional damage to a modest extent. These quantitative diffusion parameters may offer supplementary imaging evidence to support objective xerostomia evaluation in NPC patients after radiotherapy.

In addition, significant but weak associations were observed between Dmin and Dmean of the parotid gland and early changes in multiparametric functional MRI indices. Analysis of specific dosimetric metrics revealed two distinct patterns. A lower Dmin was negatively correlated with the change in FROC_D (ΔFROC_D), whereas a higher Dmean was positively correlated with increases in ΔADC, ΔDKI_D, and ΔCTRW_α. These opposing effects may reflect underlying biological mechanisms related to heterogeneous dose distributions and their differential impacts on tissue damage, repair, and microstructural reorganization.

On the one hand, a higher Dmean indicates sufficient overall radiotherapy intensity. In regions receiving an adequate radiation dose, the predominant biological responses include tissue cell death through necrosis and apoptosis, followed by tissue edema and structural disintegration. These changes lead to greater freedom of water molecule diffusion, resulting in increased values of diffusion-related parameters such as ADC, DKI_D, and CTRW_α. Previous studies revealed that a significant correlation between D values and mean dose (Dmean) is closely associated with tissue cell density, indicative of dose-dependent loss of acinar cells (31). On the other hand, insufficient Dmin was significantly associated with a reduction in ΔFROC_D. This decrease may reflect increased tissue microstructural complexity, potentially related to the survival and repair of sublethally damaged cells (32). This contrasting pattern highlights a critical issue in radiotherapy planning: treatment efficacy is determined not only by the overall magnitude of cell killing but also by the spatial uniformity of dose distribution. Therefore, future optimization of radiotherapy planning for NPC should place particular emphasis on ensuring adequate Dmin coverage across the target volume. Our preliminary findings suggest that advanced diffusion model parameters, especially metrics derived from FROC analysis, have modest potential to identify high-risk regions of gland injury at an early stage. These metrics can function only as auxiliary imaging references, and their application in biology-guided adaptive radiotherapy requires further predictive modeling, threshold optimization, and external cohort validation in future research.

However, this study has several limitations. First, our study revealed significant but weak correlations between parameter changes and radiotherapy dose. This limitation may stem from the single-center design and relatively modest sample size. Second, the assessment of xerostomia was limited to a 3-month post-radiotherapy follow-up, and MRI scans were conducted at three discrete time points. Future studies should incorporate more frequent MRI time points with long-term follow-up to better characterize the temporal evolution of PG microstructural alterations during IMRT, thereby facilitating the development of more accurate and reliable predictive models. Third, we used only subjective patient-reported xerostomia scales without salivary flow testing, and we did not perform detailed analyses of parotid functional status. Therefore, multicenter prospective trials with long-term follow-up, external validation cohorts, and objective salivary functional assessments are warranted to comprehensively clarify the correlation between diffusion imaging biomarkers and parotid gland function. Fourth, the confounding effects of chemotherapy on parotid MRI quantitative parameters and xerostomia grading could not be eliminated in the present statistical analyses. Future large-scale studies with balanced chemotherapy subgroups are needed to verify our findings after controlling for chemotherapy-related interference.

## 5 Conclusion

The findings of this study demonstrate that, in NPC patients, quantitative parameters derived from multi-b-value non-Gaussian DWI models—specifically DKI, CTRW, and FROC—enable dynamic detection of early microstructural alterations in the parotid glands following IMRT. Among these, DKI_K and CTRW_α showed statistically significant but weak correlations with xerostomia scores in patients with nasopharyngeal carcinoma. These preliminary associations indicate that the two indices may serve as exploratory imaging biomarkers for evaluating radiation-induced parotid injury; however, their reliable predictive value requires further validation. In addition, dynamic changes in ADC, DKI_D, CTRW_α, FROC_β, FROC_D, and FROC_μ were correlated with radiation dose. These findings may provide preliminary evidence to support future investigations of individualized radiotherapy regimens.

## Data Availability

The original contributions presented in the study are included in the article/**Supplementary Material**. Further inquiries can be directed to the corresponding author.

## References

[1] Lee N Puri DR Blanco AI Chao KS . Intensity-modulated radiation therapy in head and neck cancers: an update. Head Neck. (2007) 29:387–400. doi: 10.1002/hed.20332 16358297

[2] Jensen SB Vissink A Limesand KH Reyland ME . Salivary gland hypofunction and xerostomia in head and neck radiation patients. J Natl Cancer Institute Monogr. (2019) 2019.53. doi: 10.1093/jncimonographs/lgz016 31425600

[3] Zheng L Tong L Du F Ren H Xiao L . Effect of three-dimensional conformal radiotherapy and intensity-modulated radiotherapy on parotid gland function and quality of life in patients with nasopharyngeal carcinoma. Am J Trans Res. (2021) 13:5272–9. PMC8205664 34150118

[4] Martino M Fodor D Fresilli D Guiban O Rubini A Cassoni A . Narrative review of multiparametric ultrasound in parotid gland evaluation. Gland Surg. (2020) 9:2295–311. doi: 10.21037/gs-20-530 33447581 PMC7804540

[5] Liu Y Shi H Huang S Chen X Zhou H Chang H . Early prediction of acute xerostomia during radiation therapy for nasopharyngeal cancer based on delta radiomics from CT images. Quantitative Imaging Med Surg. (2019) 9:1288–302. doi: 10.21037/qims.2019.07.08 31448214 PMC6685806

[6] Chen R Xie J Chen J Li X Lin Q Xu Q . Analysis of the parotid glands on an energy spectrum CT iodine map to evaluate irradiation-induced acute xerostomia in patients with nasopharyngeal carcinoma. Technol Cancer Res Treat. (2024) 23:15330338241256814. doi: 10.1177/15330338241256814 38773777 PMC11113032

[7] Zhang Y Ou D Gu Y He X Peng W . Evaluation of salivary gland function using diffusion-weighted magnetic resonance imaging for follow-up of radiation-induced xerostomia. Korean J Radiol. (2018) 19:758–66. doi: 10.3348/kjr.2018.19.4.758 29962882 PMC6005952

[8] Trada Y Lee MT Jameson MG Chlap P Keall P Moses D . Mid-treatment 18F-FDG PET imaging changes in parotid gland correlates to radiation-induced xerostomia. Radiotherapy Oncol J Eur Soc For Ther Radiol Oncol. (2023) 186:109745. doi: 10.1016/j.radonc.2023.109745 37330056

[9] Xia X Wu L Li T Tang Q Liang L . Diffusion-weighted MRI at the late stage after radiotherapy for evaluating salivary gland injury. Curr Med Imaging. (2023) 5:20. doi: 10.2174/1573405620666230802094244 37528570

[10] Zhang X Xu Z Jin Y Huang L Wu W Gao M . Multi-parameter quantitative magnetic resonance imaging in the early assessment of radiation-induced parotid damage in patients with nasopharyngeal carcinoma following intensity-modulated radiotherapy. Oncol Lett. (2024) 27:180. doi: 10.3892/ol.2024.14313 38464343 PMC10921267

[11] Shen M Lin X Yang C Zhou Z Chen S Yin Y . Potential predictive value of IVIM MR for xerostomia in nasopharyngeal carcinoma. Radiotherapy Oncol J Eur Soc For Ther Radiol Oncol. (2024) 197:110323. doi: 10.1016/j.radonc.2024.110323 38734144

[12] Steven AJ Zhuo J Melhem ER . Diffusion kurtosis imaging: an emerging technique for evaluating the microstructural environment of the brain. AJR Am J Roentgenology. (2014) 202:W26–33. doi: 10.2214/ajr.13.11365 24370162

[13] Zhao DW Fang XM Zhou SH Luo YR Wei J Liu K . Application of diffusion kurtosis imaging in evaluating acute xerostomia in nasopharyngeal carcinoma treated with induction chemotherapy plus concurrent chemoradiotherapy. Front Oncol. (2022) 12:870315. doi: 10.3389/fonc.2022.870315 35664750 PMC9162117

[14] Sheng Y Chang H Xue K Chen J Jiao T Cui D . Characterization of prostatic cancer lesion and gleason grade using a continuous-time random-walk diffusion model at high b-values. Front Oncol. (2024) 14:1389250. doi: 10.3389/fonc.2024.1389250 38854720 PMC11157027

[15] Karaman MM Wang H Sui Y Engelhard HH Li Y Zhou XJ . A fractional motion diffusion model for grading pediatric brain tumors. NeuroImage Clin. (2016) 12:707–14. doi: 10.1016/j.nicl.2016.10.003 27761401 PMC5065039

[16] Zhou M Bao D Huang H Chen M Jiang W . Utilization of diffusion-weighted derived mathematical models to predict prognostic factors of resectable rectal cancer. Abdominal Radiol (New York). (2024) 49:3282–93. doi: 10.1007/s00261-024-04239-2 38744701

[17] Jones D . ICRU report 50—prescribing, recording and reporting photon beam therapy. Med Phys. (1994) 21. doi: 10.1118/1.597396 38306972

[18] Al AAE . ICRU report 62. Prescribing, recording and reporting photon beam therapy (supplement to ICRU report 50). Icru News. (1999) 50.

[19] Lee AW Ng WT Pan JJ Poh SS Ahn YC Alhussain H . International guideline for the delineation of the clinical target volumes (CTV) for nasopharyngeal carcinoma. Radiotherapy Oncol. (2017):S0167814017326865. doi: 10.1016/j.radonc.2017.10.032 29153464

[20] James D Cox Stetz J Pajak TF . Toxicity criteria of the Radiation Therapy Oncology Group (RTOG) and the European organization for research and treatment of cancer (EORTC). Int J Radiat Oncol Biol Phys. (1995). doi: 10.1016/0360-3016(95)00060-c 7713792

[21] Loimu V Seppälä T Kapanen M Tuomikoski L Nurmi H Mäkitie A . Diffusion-weighted magnetic resonance imaging for evaluation of salivary gland function in head and neck cancer patients treated with intensity-modulated radiotherapy. Radiotherapy Oncol J Eur Soc For Ther Radiol Oncol. (2017) 122:178–84. doi: 10.1016/j.radonc.2016.07.008 27475276

[22] Shi D Qian JJ Fan GH Shen JK Tian Y Xu L . Salivary gland function in nasopharyngeal carcinoma before and late after intensity-modulated radiotherapy evaluated by dynamic diffusion-weighted MR imaging with gustatory stimulation. BMC Oral Health. (2019) 19:288. doi: 10.1186/s12903-019-0951-x 31864328 PMC6925496

[23] Bruvo M Mahmood F . Apparent diffusion coefficient measurement of the parotid gland parenchyma. Quantitative Imaging Med Surg. (2021) 11:3812–29. doi: 10.21037/qims-20-1178 34341752 PMC8245964

[24] Dirix P De Keyzer F Vandecaveye V Stroobants S Hermans R Nuyts S . Diffusion-weighted magnetic resonance imaging to evaluate major salivary gland function before and after radiotherapy. Int J Radiat Oncol Biol Phys. (2008) 71:1365–71. doi: 10.1016/j.ijrobp.2007.12.011 18355977

[25] Astreinidou E Roesink JM Raaijmakers CP Bartels LW Witkamp TD Lagendijk JJ . 3D MR sialography as a tool to investigate radiation-induced xerostomia: feasibility study. Int J Radiat Oncol Biol Phys. (2007) 68:1310–9. doi: 10.1016/j.ijrobp.2007.01.062 17482767

[26] Jensen JH Helpern JA Ramani A Lu H Kaczynski K . Diffusional kurtosis imaging: the quantification of non-gaussian water diffusion by means of magnetic resonance imaging. Magn Reson Med. (2005) 53:1432–40. doi: 10.1002/mrm.20508 15906300

[27] Zhong J Shi P Chen Y Huang R Xiao Y Zheng X . Diffusion kurtosis imaging of a human nasopharyngeal carcinoma xenograft model: initial experience with pathological correlation. Magn Reson Imaging. (2018) 47:111–7. doi: 10.1016/j.mri.2017.12.012 29221965

[28] Chu C Wang F Zhang H Zhu Y Wang C Chen W . Whole-volume ADC histogram and texture analyses of parotid glands as an image biomarker in evaluating disease activity of primary Sjögren's syndrome. Sci Rep. (2018) 8:15387. doi: 10.1038/s41598-018-33797-x 30337659 PMC6193973

[29] Magin RL Abdullah O Baleanu D Zhou XJ . Anomalous diffusion expressed through fractional order differential operators in the Bloch-Torrey equation. J Magnetic Resonance (San Diego Calif 1997). (2008) 190:255–70. doi: 10.1016/j.jmr.2007.11.007 18065249

[30] Guichao L Xuefeng H Hai Z Xiaohong H Jianjian T Jiangui G . Diffusion-weighted magnetic resonance imaging evaluates salivary gland function damage in nasopharyngeal carcinoma patients treated with intensity-modulated radiation therapy. Cancer Res Prev Treat. (2019) 46:905–10.

[31] Marzi S Forina C Marucci L Giovinazzo G Giordano C Piludu F . Early radiation-induced changes evaluated by intravoxel incoherent motion in the major salivary glands. J Magnetic Resonance Imaging: JMRI. (2015) 41:974–82. doi: 10.1002/jmri.24626 24700435

[32] Wu VWC Ying MT Kwong DL Khong PL Wong GK Tam SY . A longitudinal study on parotid and submandibular gland changes assessed by magnetic resonance imaging and ultrasonography in post-radiotherapy nasopharyngeal cancer patients. BJR Open. (2020) 2:20200003. doi: 10.1259/bjro.20200003 33178971 PMC7583169

