# Supplementary material for: Longitudinal multi-b-value diffusion MRI for the early assessment of radiation-induced parotid injury in nasopharyngeal carcinoma
Source: Front Oncol. 2026 Jul 22;16:1896045. doi: 10.3389/fonc.2026.1896045 (PMC13437297; doi:10.3389/fonc.2026.1896045)
Supplement: Supplementary file 1 [file DataSheet1.docx]

**Supplementary results**

1.1 **Results of Consistency Test for MRI quantitative parameters**

**S**upplementary Table 1: Inter-observer consistency of quantitative parameters extracted from parotid ROIs delineated by two radiologists

| **Parameters** | **ICC** | **95%CI** | ***P*** |
| --- | --- | --- | --- |
| ADC | 0.893 | (0.749, 0.956) | ＜0.001 |
| DKI_D | 0.820 | (0.559, 0.928) | ＜0.001 |
| DKI_K | 0.873 | (0.712, 0.947) | ＜0.001 |
| CTRW_D | 0.872 | (0.689, 0.949) | ＜0.001 |
| CTRW_α | 0.830 | (0.617, 0.928) | ＜0.001 |
| CTRW_β | 0.919 | (0.691, 0.973) | ＜0.001 |
| FROC_D | 0.871 | (0.678, 0.949) | ＜0.001 |
| FROC_β | 0.897 | (0.659, 0.964) | ＜0.001 |
| FROC_μ | 0.671 | (0.335,0.855) | ＜0.001 |

The intra-class correlation coefficient (ICC) was calculated using a bidirectional random model and absolute agreement to evaluate. All parameters: D and ADC: × 10^-6^ mm^2^/s, FROC_μ: × 10^-6^ mm.

The ICC values of each parameters were between 0.671-0.919 (ADC: ICC=0.893, DKI_K: ICC=0.820, DKI_D: ICC=0.873, CTRW_D: ICC=0.872, CTRW_α: ICC=0.830, CTRW_β: ICC=0.919, FROC_D: ICC=0.871, FROC_β: ICC=0.897, FROC_μ: ICC=0.671; P＜0.005), and all the 95% CI corresponding to ICC did not contain 0, suggesting that the results are reliable and have little fluctuation. The ICC values of the parameters are generally high, indicating good consistency in the ROI delineation results.

1.2 **Results of sensitivity analysis**

Fifty-four patients were enrolled, with 54 observations each at three time points: two weeks prior to radiotherapy (Pre-RT), 15 fractions of radiotherapy (15RT), and after radiotherapy (Post-RT), totaling 324 observations. Analyze ADC, DKI_D, DKI_K, CTRW_D, CTRW_α, CTRW_β, FROC_D, FROC_β, and FROC_μ of the parotid glands at all three time points.

**S**upplementary Table 2: Comparison of left and **right** parameters for different timepoints of the treatment

| **Parameter** | **Pre-RT Mean±SD** **Left/Right** | **Pre-RT *P*** **t/W** | **15RT Mean±SD** **Left/Right** | **15RT *P*** **t/W** | **Post-RT Mean±SD**  **Left/Right** | **Post-RT *P*** **t/W** |
| --- | --- | --- | --- | --- | --- | --- |
| ADC | 896.91±132.88 897.24±153.45 | 0.988 0.550 | 1089.97±188.39 1114.74±192.55 | 0.244 0.329 | 1271.75±219.41 1263.36±220.91 | 0.648 0.918 |
| DKI_D | 1124.04±202.57 1147.32±202.33 | 0.463 0.084 | 1430.77±237.02 1491.38±259.46 | 0.081 0.097 | 1664.21±264.26 1704.42±237.56 | 0.221 0.137 |
| DKI_K | 921.49±194.14 1034.62±382.10 | 0.023 0.021 | 836.57±217.41 910.58±263.80 | 0.033 0.019 | 761.55±209.75 858.27±294.56 | 0.007 <0.001 |
| CTRW_D | 948.62±155.05 949.56±176.29 | 0.970 0.392 | 1208.22±196.48 1249.90±203.51 | 0.106 0.137 | 1411.03±221.65 1418.52±211.27 | 0.745 0.473 |
| CTRW_α | 748.12±77.35 736.68±86.02 | 0.214 0.333 | 803.19±91.57 783.05±105.35 | 0.076 0.147 | 841.66±77.46 807.90±98.43 | 0.009 0.011 |
| CTRW_β | 778.90±59.02 749.33±65.39 | <0.001 <0.001 | 760.40±80.43 742.28±81.77 | 0.123 0.157 | 760.72±70.51 741.36±79.68 | 0.045 0.059 |
| FROC_D | 685.67±113.95 657.27±137.90 | 0.089 0.093 | 848.93±173.65 838.23±182.26 | 0.535 0.407 | 1003.91±211.96 966.26±216.63 | 0.045 0.075 |
| FROC_β | 754.88±53.97 724.89±64.55 | <0.001 <0.001 | 747.08±66.61 727.13±67.08 | 0.036 0.040 | 753.21±65.65 736.11±73.48 | 0.049 0.095 |
| FROC_μ | 3008.72±239.00  3048.02±318.47 | 0.437 0.567 | 3147.35±303.71 3162.39±298.98 | 0.734 0.746 | 3188.07±330.36 3153.73±355.48 | 0.514 0.431 |

Within the same mean cell, the first row is on the left, and the second row is on the right; The first row of column P pairs t to test bilateral P values, and the second row tests the Wilcoxon symbolic rank for precise P values.All parameters: D and ADC: × 10^-6^ mm^2^/s, FROC_μ: × 10^-6^ mm.

Supplementary Table 2 used individual patients as matched units to compare 9 parameters of the left and right parotid glands within each patient across three time points. At two weeks prior to radiotherapy, significant lateral differences were observed in DKI_K, CTRW_β, and FROC_β: DKI_K was higher on the right, while CTRW_β and FROC_β were higher on the left. At 15 fractions of radiotherapy, DKI_K and FROC_β still showed lateral differences with the same directional bias as pre-radiotherapy measurements. After radiotherapy, lateral differences were observed in DKI_K, CTRW_α, CTRW_β, FROC_D, and FROC_β; DKI_K remained elevated on the right side, whereas the other four parameters showed higher values on the left side. Wilcoxon test results provided consistent robust evidence for DKI_K and CTRW_α, while associations for CTRW_β, FROC_D, and FROC_β were borderline non-significant. No consistent lateral disparities were identified for ADC, DKI_D, CTRW_D, and FROC_μ. These findings demonstrate systematic lateral heterogeneity across several imaging parameters, highlighting the need for subsequent unilateral sensitivity analyses.

Supplementary Table 3: Sensitivity analysis of temporal effects based on left parotid gland data

| **Parameter** | **Pre-RT Mean(SE)** | **15RT**  **Mean(SE)** | **Post-RT**  **Mean(SE)** | **P^1^** | **15RT-Pre**  **Difference(P^2^)** | **Post-Pre**  **Difference(P^3^)** | **Post-15RT**  **Difference(P^4^)** |
| --- | --- | --- | --- | --- | --- | --- | --- |
| ADC | 896.91(25.01) | 1089.97(25.01) | 1271.75(25.01) | <0.001 | 193.05(<0.001) | 374.84(<0.001) | 181.78(<0.001) |
| DKI_D | 1124.04(32.11) | 1430.77(32.11) | 1664.21(32.11) | <0.001 | 306.73(<0.001) | 540.16(<0.001) | 233.43(<0.001) |
| DKI_K | 921.49(28.21) | 836.57(28.21) | 761.55(28.21) | <0.001 | -84.92(0.026) | -159.94(<0.001) | -75.02(0.061) |
| CTRW_D | 948.62(26.27) | 1208.22(26.27) | 1411.03(26.27) | <0.001 | 259.60(<0.001) | 462.40(<0.001) | 202.80(<0.001) |
| CTRW_α | 748.12(11.21) | 803.19(11.21) | 841.66(11.21) | <0.001 | 55.07(<0.001) | 93.54(<0.001) | 38.47(0.003) |
| CTRW_β | 778.90(9.60) | 760.40(9.60) | 760.72(9.60) | 0.130 | -18.50(0.233) | -18.19(0.249) | 0.32(1.000) |
| FROC_D | 685.67(23.32) | 848.93(23.32) | 1003.91(23.32) | <0.001 | 163.26(<0.001) | 318.24(<0.001) | 154.98(<0.001) |
| FROC_β | 754.88(8.48) | 747.08(8.48) | 753.21(8.48) | 0.667 | -7.80(1.000) | -1.67(1.000) | 6.13(1.000) |
| FROC_μ | 3008.72(39.95) | 3147.35(39.95) | 3188.07(39.95) | 0.001 | 138.63(0.019) | 179.34(0.001) | 40.71(1.000) |

The mean in the table is the marginal mean of the mixed effects model; the values in parentheses are standard errors; Pairwise comparisons are given by the difference and Bonferroni correction P-value, with the difference being the latter time point minus the previous one. *P^1^*: overall main effect of time; *P^2^*: Pre-radiotherapy and after 15 fractions of radiotherapy; *P^3^*: Pre-radiotherapy and the end of radiotherapy; *P^4^*: After 15 fractions of radiotherapy and the end of radiotherapy. All parameters: D and ADC: × 10^-6^ mm^2^/s, FROC_μ: × 10^-6^ mm.

Supplementary Table 3 included only data from the left parotid gland, and a mixed-effects model with patient-specific random intercepts was applied to assess the temporal effects. ADC, DKI_D, CTRW_D, CTRW_α, FROC_D, and FROC_μ increased over time, with statistically significant overall temporal effects for all these parameters. DKI_K decreased over time; significant differences were found between pre-radiotherapy and after 15 fractions of radiotherapy, as well as between pre-radiotherapy and post-radiotherapy completion, whereas the difference between after 15 fractions of radiotherapy and the end of radiotherapy was borderline significant. ADC, DKI_D, CTRW_D, CTRW_α, and FROC_D showed significant differences across all three pairwise comparisons, and FROC_μ predominantly exhibited a progressive elevation throughout the treatment course. The overall temporal effects of CTRW_β and FROC_β failed to reach statistical significance, and no consistent intergroup differences were observed in the three pairwise comparisons after Bonferroni correction. The model based solely on left parotid data revealed distinct temporal variations in most diffusion- and fractal-related metrics across the three time points, with change directions consistent with those anticipated from the full-cohort analysis. The primary temporal trends were preserved after excluding right parotid gland data separately, indicating that the study conclusions were robust in the lateral sensitivity analysis.

Supplementary Table 4 Sensitivity analysis of temporal effects based on the right parotid gland

| **Parameter** | **Pre-RT Mean(SE)** | **15RT**  **Mean(SE)** | **Post-RT**  **Mean(SE)** | **P^1^** | **15RT-Pre**  **Difference(P^2^)** | **Post-Pre**  **Difference(P^3^)** | **Post-15RT**  **Difference(P^4^)** |
| --- | --- | --- | --- | --- | --- | --- | --- |
| ADC | 897.24(25.99) | 1114.74(25.99) | 1263.36(25.99) | <0.001 | 217.50(<0.001) | 366.12(<0.001) | 148.62(<0.001) |
| DKI_D | 1147.32(31.88) | 1491.38(31.88) | 1704.42(31.88) | <0.001 | 344.06(<0.001) | 557.09(<0.001) | 213.03(<0.001) |
| DKI_K | 1034.62(43.20) | 910.58(43.20) | 858.27(43.20) | <0.001 | -124.04(0.017) | -176.34(<0.001) | -52.30(0.728) |
| CTRW_D | 949.56(26.89) | 1249.90(26.89) | 1418.52(26.89) | <0.001 | 300.35(<0.001) | 468.96(<0.001) | 168.61(<0.001) |
| CTRW_α | 736.68(13.19) | 783.05(13.19) | 807.90(13.19) | <0.001 | 46.37(<0.001) | 71.21(<0.001) | 24.84(0.102) |
| CTRW_β | 749.33(10.34) | 742.28(10.34) | 741.36(10.34) | 0.698 | -7.05(1.000) | -7.97(1.000) | -0.92(1.000) |
| FROC_D | 657.27(24.74) | 838.23(24.74) | 966.26(24.74) | <0.001 | 180.96(<0.001) | 308.99(<0.001) | 128.03(<0.001) |
| FROC_β | 724.89(9.32) | 727.13(9.32) | 736.11(9.32) | 0.423 | 2.24(1.000) | 11.21(0.645) | 8.97(0.964) |
| FROC_μ | 3048.02(44.25) | 3162.39(44.25) | 3153.73(44.25) | 0.081 | 114.37(0.133) | 105.71(0.189) | -8.66(1.000) |

The mean in the table is the marginal mean of the mixed effects model; the values in parentheses are standard errors; Pairwise comparisons are given by the difference and Bonferroni correction P-value, with the difference being the latter time point minus the previous one. *P^1^*: overall main effect of time; *P^2^*: Pre-radiotherapy and after 15 fractions of radiotherapy; *P^3^*: Pre-radiotherapy and the end of radiotherapy; *P^4^*: After 15 fractions of radiotherapy and the end of radiotherapy. All parameters: D and ADC: × 10^-6^ mm^2^/s, FROC_μ: × 10^-6^ mm.

Supplementary Table 4 included only data from the right parotid gland, using the same model structure as for the left parotid gland. ADC, DKI_D, CTRW_D, CTRW_α, and FROC_D increased over time, presenting statistically significant overall temporal effects. DKI_K declined with treatment progression; significant differences were maintained between pre-radiotherapy and after 15 fractions of radiotherapy, as well as between pre-radiotherapy and post-radiotherapy completion, whereas no significant difference was detected between the 15 fractions of radiotherapy and the end of radiotherapy. ADC, DKI_D, CTRW_D, and FROC_D yielded significant results in all three pairwise comparisons, and the statistical significance of CTRW_α was mainly driven by the differences between pre-radiotherapy and after 15 fractions of radiotherapy, and between pre-radiotherapy and post-radiotherapy completion. No significant temporal effects were observed for CTRW_β and FROC_β. The overall P-value of FROC_μ was 0.081, indicating weak temporal alterations in the right parotid gland data, and none of the corrected pairwise comparisons reached statistical significance. The right-sided and left-sided models exhibited largely consistent changes in direction and statistical significance for ADC, DKI_D, DKI_K, CTRW_D, CTRW_α, and FROC_D.

Supplementary Table 5: Sensitivity analysis of temporal effects based on patient-level averaged bilateral parotid gland measurements

| **Parameter** | **Pre-RT Mean(SE)** | **15RT**  **Mean(SE)** | **Post-RT**  **Mean(SE)** | **P^1^** | **15RT-Pre**  **Difference(P^2^)** | **Post-Pre**  **Difference(P^3^)** | **Post-15RT**  **Difference(P^4^)** |
| --- | --- | --- | --- | --- | --- | --- | --- |
| ADC | 897.08(23.40) | 1102.35(23.40) | 1267.55(23.40) | <0.001 | 205.28(<0.001) | 370.48(<0.001) | 165.20(<0.001) |
| DKI_D | 1135.68(27.50) | 1461.08(27.50) | 1684.31(27.50) | <0.001 | 325.40(<0.001) | 548.63(<0.001) | 223.23(<0.001) |
| DKI_K | 978.05(30.68) | 873.58(30.68) | 809.91(30.68) | <0.001 | -104.48(<0.001) | -168.14(<0.001) | -63.66(0.064) |
| CTRW_D | 949.09(23.67) | 1229.06(23.67) | 1414.77(23.67) | <0.001 | 279.97(<0.001) | 465.68(<0.001) | 185.71(<0.001) |
| CTRW_α | 742.40(10.94) | 793.12(10.94) | 824.78(10.94) | <0.001 | 50.72(<0.001) | 82.38(<0.001) | 31.66(0.002) |
| CTRW_β | 764.12(8.69) | 751.34(8.69) | 751.04(8.69) | 0.204 | -12.78(0.381) | -13.08(0.355) | -0.30(1.000) |
| FROC_D | 671.47(22.43) | 843.58(22.43) | 985.09(22.43) | <0.001 | 172.11(<0.001) | 313.62(<0.001) | 141.51(<0.001) |
| FROC_β | 739.89(7.80) | 737.11(7.80) | 744.66(7.80) | 0.588 | -2.78(1.000) | 4.77(1.000) | 7.55(0.924) |
| FROC_μ | 3028.37(34.32) | 3154.87(34.32) | 3170.90(34.32) | <0.001 | 126.50(0.007) | 142.53(0.002) | 16.03(1.000) |

The mean in the table is the marginal mean of the mixed effects model; the values in parentheses are standard errors; Pairwise comparisons are given by the difference and Bonferroni correction P-value, with the difference being the latter time point minus the previous one. *P^1^*: overall main effect of time; *P^2^*: Pre-radiotherapy and after 15 fractions of radiotherapy; *P^3^*: Pre-radiotherapy and the end of radiotherapy; *P^4^*: After 15 fractions of radiotherapy and the end of radiotherapy. All parameters: D and ADC: × 10^-6^ mm^2^/s, FROC_μ: × 10^-6^ mm.

Supplementary Table 5 presented data derived by averaging measurements of the left and right parotid glands from the same patient, yielding a total of 162 patient-level records across three time points for 54 patients. Mixed-effects models with random patient intercepts were used to assess time effects. Patient-averaged analysis showed significant overall temporal increases in ADC, DKI_D, CTRW_D, CTRW_α, FROC_D, FROC_μ, and a reduction in DKI_K. Adjusted pairwise contrasts showed full three-group significance for ADC, DKI_D, CTRW_D, and CTRW_α; DKI_K showed a borderline Post vs 15RT difference, and FROC_μ rose significantly from Pre to 15RT and from Pre to Post. CTRW_β and FROC_β showed no significant time effect. Consistent trends between averaged and unilateral models confirmed robust primary findings after adjusting for bilateral parotid correlation.

**1.3 Results of FDR-Corrected Multiple Comparison**

All pairwise time-point comparisons and inter-temporal change comparisons were corrected via the Benjamini–Hochberg FDR method. Repeated-measures ANOVA test was used for normally distributed differences, and Wilcoxon signed-rank tests for non-normal differences.

Supplementary Table 6: FDR-Corrected Multiple Comparison for different timepoints of the treatment

| **Parameter** | **Pre** | **15RT** | **Post** | ***q1*** | ***q2*** | ***q3*** |
| --- | --- | --- | --- | --- | --- | --- |
| ADC | 897.08±142.86 | 1102.35±189.99 | 1267.55±219.17 | <0.001ᵇ | <0.001ᵇ | <0.001ᵃ |
| DKI_D | 1135.68±201.84 | 1461.07±249.19 | 1684.31±250.90 | <0.001ᵇ | <0.001ᵇ | <0.001ᵃ |
| DKI_K | 916.07(284.73) | 812.68(237.61) | 742.93(192.89) | <0.001ᵇ | <0.001ᵇ | <0.001ᵇ |
| CTRW_D | 949.09±165.23 | 1229.06±200.18 | 1414.77±215.54 | <0.001ᵇ | <0.001ᵇ | <0.001ᵃ |
| CTRW_α | 742.40±81.62 | 814.19(157.61) | 832.41(114.79) | <0.001ᵃ | <0.001ᵇ | <0.001ᵇ |
| CTRW_β | 764.12±63.75 | 751.34±81.24 | 767.41(97.416) | 0.082ᵇ | 0.082ᵇ | 0.969ᵇ |
| FROC_D | 671.47±126.71 | 843.58±177.26 | 1017.24(285.70) | <0.001ᵇ | <0.001ᵇ | <0.001ᵇ |
| FROC_β | 739.89±61.10 | 737.11±67.28 | 757.25(86.05) | 0.621ᵃ | 0.511ᵇ | 0.317ᵇ |
| FROC_μ | 3028.98(278.41) | 3154.87±300.04 | 3170.90±341.98 | <0.001ᵇ | 0.001ᵇ | 0.710ᵃ |

Normally and non-normally distributed data were presented as mean±SD and median (IQR), respectively. *q*-values represent FDR-adjusted *P* values derived from the Benjamini–Hochberg procedure. *q₁*, comparison between 15RT and Pre-RT; *q₂*, comparison between Post-RT and Pre-RT; *q₃,* comparison between Post-RT and 15RT. (a: Repeated-measures ANOVA; b: Wilcoxon signed-rank test)

Supplementary Table 6 compared quantitative MRI parameters across all 108 parotid gland samples, with the Benjamini–Hochberg false discovery rate (FDR) method used to control for type I error due to multiple testing. FDR-adjusted pairwise comparisons showed persistent significant differences in ADC, DKI_D, DKI_K, CTRW_D, CTRW_α, and FROC_D across all three time-point contrasts, with consistent rising trends for ADC, DKI_D, CTRW_D, CTRW_α, and FROC_D, and a sustained reduction in DKI_K matching the original analysis. CTRW_β and FROC_β showed no significant temporal differences, suggesting weak sensitivity to radiation-related changes. FROC_μ was significantly higher at 15RT and Post relative to Pre, with no difference between 15RT and Post, implying its capacity to capture only early and mid-treatment shifts. The FDR correction preserved the primary positive conclusions while reducing the false-positive rate.

Supplementary Table 7: FDR-Corrected Multiple Comparison of parameter dynamic changes for different timepoints of the treatment

| **Parameter** | **15RT-Pre** | **Post-Pre** | **Post-15RT** | ***q^1^*** | ***q^2^*** | ***q^3^*** |
| --- | --- | --- | --- | --- | --- | --- |
| ΔADC | 205.28±181.76 | 397.24(290.43) | 165.20(290.43) | <0.001ᵇ | 0.124ᵇ | <0.001ᵇ |
| ΔDKI_D | 325.40±258.65 | 548.63±293.91 | 226.34(251.20) | <0.001ᵇ | 0.023ᵇ | <0.001ᵇ |
| ΔDKI_K | -116.11(265.98) | -150.37(239.69) | -64.23(161.57) | <0.001ᵇ | 0.102ᵇ | <0.001ᵇ |
| ΔCTRW_D | 279.97(161.57) | 465.68±229.05 | 181.07(190.87) | <0.001ᵇ | 0.002ᵇ | <0.001ᵇ |
| ΔCTRW_α | 51.88(113.23) | 82.20(92.95) | 31.66(92.95) | <0.001ᵇ | 0.218ᵇ | <0.001ᵇ |
| ΔCTRW_β | -6.01(93.40) | -13.08±73.47 | -0.30±81.97 | 0.969ᵇ | 0.387ᵇ | 0.101ᵃ |
| ΔFROC_D | 187.38(179.49) | 313.62±178.75 | 142.81(143.82) | <0.001ᵃ | 0.101ᵇ | <0.001ᵇ |
| ΔFROC_β | 0.34(74.98) | 4.77±66.09 | 7.55±70.80 | 0.326ᵇ | 0.326ᵇ | 0.621ᵃ |
| ΔFROC_μ | 170.93(425.14) | 142.53±423.45 | 16.035(425.1) | 0.710ᵇ | 0.102ᵇ | <0.001ᵃ |

Normally and non-normally distributed data were presented as mean±SD and median (IQR), respectively. *q*-values represent FDR-adjusted *P* values derived from the Benjamini–Hochberg procedure. *q₁*, comparison between 15RT and Pre-RT; *q₂*, comparison between Post-RT and Pre-RT; *q₃,* comparison between Post-RT and 15RT. (a: Repeated-measures ANOVA test; b: Wilcoxon signed-rank test)

Supplementary Table 7 compared the magnitudes of dynamic changes in MRI parameters across three treatment phases to test for consistent change rates across stages. FDR correction demonstrated that the changes between the end of radiotherapy and pre-radiotherapy (ΔADC, Δ DKI_D, ΔDKI_K, ΔCTRW_D, ΔCTRW_α, and ΔFROC_D) differ significantly from those between the end of radiotherapy and after 15 fractions of radiotherapy, indicating that the cumulative changes are significantly stronger than those in the short-range changes. The Δ ADC, Δ CTRW_α, and Δ FROC_D between 15 fractions of radiotherapy and pre-radiotherapy did not significantly differ from the changes between the end of radiotherapy and after 15 fractions of radiotherapy, suggesting that the amplitudes of early and late changes were similar; Δ DKI_D and Δ CTRW_D remained significant in this comparison, indicating differences in the rate of change in their stages. Δ DKI_K is a negative change, with the largest decline between the end of radiotherapy and pre-radiotherapy, consistent with the downward trend compared to the original timeline. ΔCTRW_β, ΔFROC_β, and ΔFROC_μ were not significant in these comparisons, indicating weak stability over periodic changes. This table addresses multiple comparison issues at the level of change and supports the robustness of major dynamic conclusions.
